# Anti-Inflammatory Effects of *Clematis terniflora* Leaf on Lipopolysaccharide-Induced Acute Lung Injury

**DOI:** 10.1155/2024/6653893

**Published:** 2024-01-09

**Authors:** Ji-Yeong Park, Min-Jong Kim, Young-Ae Choi, Yeon-Yong Kim, Soyoung Lee, Jae-Min Chung, Sang-Yong Kim, Gil-Saeng Jeong, Sang-Hyun Kim

**Affiliations:** ^1^Cell and Matrix Research Institute, Department of Pharmacology, School of Medicine, Kyungpook National University, Daegu 41944, Republic of Korea; ^2^Functional Biomaterial Research Center, Korea Research Institute of Bioscience and Biotechnology, Jeongeup 56212, Republic of Korea; ^3^Department of Gardens and Education, Korea National Arboretum, Pocheon 11186, Republic of Korea; ^4^DMZ Botanic Garden, Korea National Arboretum, Yanggu 24564, Republic of Korea; ^5^College of Pharmacy, Chungnam National University, Daejeon 34134, Republic of Korea

## Abstract

For centuries, natural products are regarded as vital medicines for human survival. *Clematis terniflora* var. *mandshurica* (Rupr.) Ohwi is an ingredient of the herbal medicine, Wei Ling Xian, which has been used in Chinese medicine to alleviate pain, fever, and inflammation. In particular, *C. terniflora* leaves have been used to cure various inflammatory diseases, including tonsillitis, cholelithiasis, and conjunctivitis. Based on these properties, this study aimed to scientifically investigate the anti-inflammatory effect of an ethanol extract of leaves of *C. terniflora* (EELCT) using activated macrophages that play central roles in inflammatory response. In this study, EELCT inhibited the essential inflammatory mediators, such as nitric oxide, cyclooxygenase-2, tumor necrosis factor-*α*, interleukin- (IL-) 6, IL-1*β*, and inducible nitric oxide synthase, by suppressing the nuclear factor-*κ*B and mitogen-activated protein kinase activation in macrophages. Acute lung injury (ALI) is a fatal respiratory disease accompanied by serious inflammation. With high mortality rate, the disease has no effective treatments. Therefore, new therapeutic agents must be developed for ALI. We expected that EELCT can be a promising therapeutic agent for ALI by reducing inflammatory responses and evaluated its action in a lipopolysaccharide- (LPS-) induced ALI model. EELCT alleviated histological changes, immune cell infiltration, inflammatory mediator production, and protein-rich pulmonary edema during ALI. Collectively, our results may explain the traditional usage of *C. terniflora* in inflammatory diseases and suggest the promising potential of EELCT as therapeutic candidate for ALI.

## 1. Introduction

Plants are essential elements for treating various diseases in oriental medicine. *Clematis* is a herb genus containing approximately 300 species in the family Ranunculaceae [1]. Wei Ling Xian, which comes from the roots and rhizomes of several *Clematis* species, is prescribed as an analgesic, antipyretic, anticancer, and anti-inflammatory agent in the ancient books of East Asia, including Chinese Pharmacopeia and Donguibogam (Korean traditional ancient book completed in 1613 by Jun Heo) [2, 3]. One particular plant, *Clematis terniflora* var. *mandshurica* (Rupr.) Ohwi, is mainly distributed in China, Japan, and Korea. It is known as “Earri” or “Wi Ryeong Seon” in Korea and also has been used to address pain and fever issues in oriental medicine [4, 5]. In particular, the leaf of *C. terniflora* is described as treatment for inflammation-related diseases, such as tonsillitis, cholelithiasis, and conjunctivitis in Traditional Chinese Medicine Dictionary [6].

Inflammation is the first defense response of the innate immune system to protecting the body against infections and injuries. However, excessive inflammation causes enormous tissue damage and multiple disorders [7]. Macrophages play important roles in inflammatory processes by secreting diverse inflammatory mediators, including nitric oxide (NO), cyclooxygenase (COX)-2, tumor necrosis factor- (TNF-) *α*, interleukin- (IL-) 6, IL-1*β*, and inducible nitric oxide synthase (iNOS) [8]. The expression of these mediators is associated with life-threatening inflammatory disorders, e.g., sepsis-related multiple organ dysfunction/multiple organ failure, neurodegenerative disorders, microbial infections, and acute lung injury (ALI) [9]. Inflammatory mediators are regulated by intracellular signaling pathways, in particular, nuclear factor- (NF-) *κ*B, which is a pleiotropic transcription factor active during innate immunity and inflammation responses [10, 11]. Mitogen-activated protein kinases (MAPKs) are serine/threonine protein kinases, which phosphorylate substrates, to activate or deactivate target proteins, specifically in macrophages [12]. Consecutive phosphorylation triggers MAPK cascades, which have important roles in the pathogenesis and development of inflammation [13].

ALI is a pulmonary disease characterized by excessive inflammatory responses and dysfunction of the respiratory tract. ALI can be caused by both direct and indirect lung damage, including sepsis, aspiration pneumonia, inhalation injury, pancreatitis, and blood transfusions [14]. Whatever etiology causes, the ALI pathogenesis is attributed to out-of-control inflammatory response. As irreversible inflammation and pulmonary damage can lead to death, it is recognized as a serious public health problem [15]. ALI treatments can be divided into supportive care and pharmacological interventions (corticosteroids, anti-inflammatory agents, and antioxidants); however, these therapies cannot effectively cure ALI and improve patients' quality of life [16, 17]. Therefore, it is urgent to develop new drugs for ALI. As Gram-negative bacterial infection is one of the most common causes of ALI, the intratracheal injection of lipopolysaccharide (LPS) into animals is typically considered as a well-established experimental model, which imitates human ALI [18, 19]. In this model, LPS induces several inflammatory symptoms, including tissue damage, protein-rich edema, immune cell infiltration, and increased inflammatory mediators in the lung [20].

The study of traditional medicinal plants is considered an attractive means for developing promising complementary and alternative medicine in current health problems. Accordingly, we examined the mechanism action of ethanol extract of leaves of *C. terniflora* (EELCT) in inflammatory responses using LPS-stimulated macrophages. Moreover, we expected that EELCT has a therapeutic possibility against inflammatory diseases and identified the anti-inflammatory activity in an LPS-induced ALI model.

## 2. Materials and Methods

### 2.1. Reagents

Reagents were obtained from the following suppliers: Dulbecco's modified Eagle's medium (DMEM) (12800-017) and fetal bovine serum (FBS) (16000-044) from Gibco, Grand Island, NY. Antibiotic-antimycotic solution (SV30079.01) from HyClone Laboratories, Logan, UT. LPS from *Escherichia coli* 055:B5 (L2637), dexamethasone (Dex) (D4902), sodium nitrite (S2252), sulfanilamide (S9251), and *N*-(1-naphthyl)-ethylenediamine dihydrochloride (N9125) from Sigma-Aldrich, St. Louis, MO.

### 2.2. EELCT Preparation and Identification

**Figure 1:**
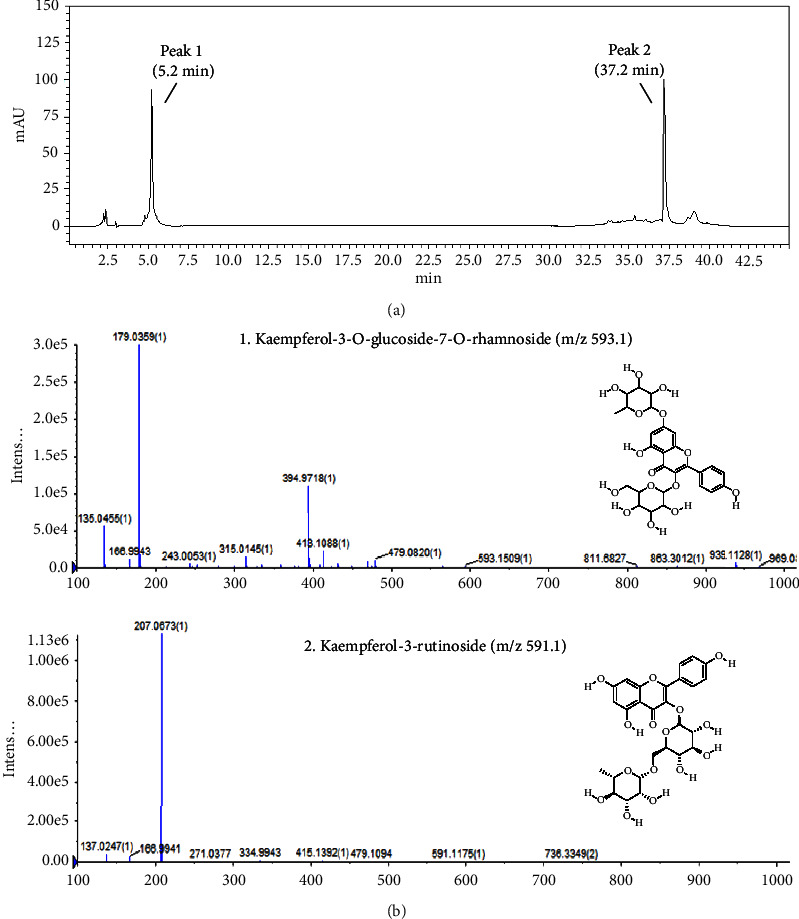
HPLC profiling of EELCT. (a) HPLC-DAD analysis was performed on Capcell Pak C18 column (5 *μ*m × 4.6 mm × 250 mm). The main peaks were detected from the chromatogram. (b) The peaks were identified as kaempferol-3-O-glucoside-7-O-rhamnoside and kaempferol-3-rutinoside by mass spectrum.

*Clematis terniflora* var. *mandshurica* (Rupr.) Ohwi was obtained from the Korea National Arboretum in Pocheon, Republic of Korea (voucher specimen no. JMC15119). Material (30 kg) was ground and extracted in ethanol at 70°C for 5 h twice in water bath. Then, the extract was filtered, lyophilized, and then stored at 4°C. The dried extract yield was approximately 5.3% from starting crude materials. To identify individual components in EELCT, we used high-performance liquid chromatography (HPLC) as previously reported [21]. Analyses were performed using Agilent HP 1260 Infinity Series liquid chromatograph (Agilent Technologies, Santa Clara, CA) with a Capcell Pak C18 column (5 *μ*m × 4.6 mm × 250 mm; Shiseido, Japan) and Agilent 6120 single quadrupole. Chromatography was performed at room temperature at a flow rate of 1 mL/min, and a 10 *μ*L sample was analyzed over 120 min. The mobile phase consisted of 0.1% formic acid (A) and acetonitrile (B) in a ratio specified by the following binary gradient with linear interpolation: 0 min 20% B, 60 min 30% B, 70 min 60% B, 100 min 70% B, and 120 min 20% B. UV spectra were collected using a diode array detector system (DAD) (Agilent Technologies) every 0.4 s from 260 nm to 345 nm with a resolution of 2 nm. Mass spectrometry was carried out using Agilent 6120 single quadrupole (Agilent Technologies) and analyzed using Mass Hunter Qualitative Analysis Software Version B.06.00 (Agilent Technologies). The analyses were performed under the following conditions: capillary voltage, 3.2 kV; sample cone voltage, 27 V; source temperature, 100°C; gas flow, 600 L/h; and gas temperature, 40°C. The mass scan range was 50–1000 m/z. Raw data were processed based on retention time and characteristic behavior of MS, including the exact mass, quasi-molecular ions, and in-source fragmentation. These data were compared with those of known compounds from an in-house plant database and existing literature. These data are shown in Figure 1.

### 2.3. Cell Culture and Measurement of NO Production

RAW 264.7 cells were grown in DMEM supplemented with heat-inactivated 10% FBS and antibiotic-antimycotic solution in an atmosphere of 5% CO_2_ at 37°C. In this study, Dex (3.925 *μ*g/mL; 10 *μ*M), a typical corticosteroid medicine for inflammation, was used as a positive control [22]. To measure NO production, RAW 264.7 cells (1 × 10^5^ cells/well in a 96-well plate) were pretreated with EELCT (1, 10, or 100 *μ*g/mL) or Dex (10 *μ*M) for 1 h and stimulated with LPS (100 ng/mL). After 24 h, supernatants were collected and centrifuged at 400 g for 5 min at 4°C. NO concentration was measured using Griess reagent consisting of 1% sulfanilamide, 0.1% N-[1-naphthyl]-ethylenediamine dihydrochloride, and 2.5% phosphoric acid. In a 96-well plate, equal volumes of culture media and Griess reagent were incubated for 5 min at room temperature. The absorbance was measured at 540 nm using a spectrophotometer (Molecular Devices). A standard curve using serial dilutions of sodium nitrite was constructed, and the amount of NO was calculated.

### 2.4. RNA Extraction and qPCR

RAW 264.7 cells (5 × 10^5^ cells/well in a 12-well plate) were pretreated with EELCT (1, 10, or 100 *μ*g/mL) or Dex (10 *μ*M) for 1 h and stimulated with LPS (100 ng/mL) for 6 h. Before qPCR, total RNA was extracted using RNAiso Plus (Takara Bio Inc., Shiga, Japan) and quantified using a NanoDrop 2000 spectrophotometer (Thermo Fisher Scientific, Wilmington, MA). cDNA was then synthesized from 1 *μ*g of total RNA using a RevertAid First Strand cDNA Synthesis Kit (Thermo Fisher Scientific) according to the manufacturer's protocol. qPCR was conducted using an Applied Biosystems StepOnePlus™ Real-time PCR System (Life Technologies Co., Kallang Avenue, Singapore) according to the manufacturer's protocol. In brief, 1 *μ*L of cDNA, 1 *μ*L of each sense and antisense primer solution (0.5 *μ*M), 10 *μ*L of QGreenBlue Master Mix High ROX (QBHR-05 2X, Thermo Fisher Scientific), and 7 *μ*L of nuclease-free water were mixed in reaction tubes. Relative quantification of target gene was calculated using the 2−^∆∆Cq^ method in StepOnePlus PCR system software (Thermo Fisher Scientific). Primer sequences used in this study are shown in Supplementary Table S1.

### 2.5. ELISA

Inflammatory cytokine levels in BALF and culture media were measured by ELISA. RAW 264.7 cells (5 × 10^5^ cells/well in a 12-well plate) were pretreated with EELCT (1, 10, and 100 *μ*g/mL) and Dex (10 *μ*M) for 1 h and stimulated with LPS (100 ng/mL) for 12 h or 24 h. ELISA was performed on a 96-well immune plate (Nunc, Rochester, NY) using specific kits according to the manufacturer's instructions. The following kits were used: TNF-*α* and IL-6 (BD Biosciences, San Diego, CA); IL-1*β* (Invitrogen, Carlsbad, CA); and myeloperoxidase (MPO) (R&D Systems, Minneapolis, MN). The absorbance was measured at 450 nm using a spectrophotometer (Molecular Devices).

### 2.6. Western Blot

The protein levels in lung tissues and macrophages were determined using Western blot. RAW 264.7 cells (1.5 × 10^6^ cells/well in a 6-well plate) were pretreated with EELCT (1, 10, and 100 *μ*g/mL) and Dex (10 *μ*M) for 1 h and stimulated with LPS (100 ng/mL) for 15 min or 12 h. To obtain total protein extract, cells and tissues were lysed in radioimmunoprecipitation assay buffer (50 mM Tris, 150 mM NaCl, 1% Triton X-100, 0.1% SDS, 0.5% Na-deoxycholate, 0.1 M DTT, and 1 mM Na_3_VO_4_) plus a protease and phosphatase inhibitor cocktail (Roche Diagnostics, Indianapolis, IN). Lysates were centrifuged at 15,000 *g* for 20 min at 4°C. Equal amounts of total protein quantities were separated by sodium dodecyl sulfate-polyacrylamide gel electrophoresis on an 8.5% gel and transferred to nitrocellulose membranes. After blocking with 5% bovine serum albumin in Tris-buffered saline containing 0.5% Tween 20, membranes were incubated overnight with target primary antibodies and then with appropriate anti-IgG horseradish peroxidase-conjugated secondary antibodies. All antibodies used for Western blot are listed in Supplementary Table S2.

### 2.7. LPS-Induced ALI Model

To investigate the anti-inflammatory effect of EELCT *in vivo*, we used an LPS-induced ALI model characterized by serious inflammatory response [23]. BALB/c mice (male, 6 weeks old, and body weight: 19–21 g) were purchased from the Dae-Han Experimental Animal Center in Daejeon, Republic of Korea. Animals were housed at a temperature of 22°C ± 1°C, humidity of 55% ± 5%, light/dark cycle of 12 h, and air exchange rate of 15 min/h. Feed and water were supplied *ad libitum*. Animal experiments were conducted in accordance with Public Health Service Policy on the Humane Care and Use of Laboratory Animals Guidelines and approved by the Institutional Animal Care and Use Committee of Kyungpook National University (IRB #2022-0389).

First, 30 mice were randomly divided into six groups: control; ALI; ALI and 0.1, 1, and 10 mg/kg EELCT; and 2 mg/kg Dex. The dosages were selected based on our preliminary experiments. Mice were intraperitoneally injected with a phosphate-buffered saline (PBS) : ketamine : Rompun (7 : 2 : 1) mixture anesthetic and intratracheally injected with 50 *μ*L of LPS (5 mg/kg). An equal volume of PBS was injected into control group. EELCT and Dex were orally administrated 1 h before ALI induction. After 24 h, mice were humanely sacrificed by anesthetic overdose and lung tissue and bronchoalveolar lavage fluid (BALF) were collected for analysis.

### 2.8. Histopathological Evaluations

The left lung lobe was excised and fixed in 4% paraformaldehyde. After 72 h, tissues were embedded in paraffin and stained with hematoxylin and eosin (H&E) [24]. Pathological changes were observed under light microscopy (PerkinElmer, Waltham, MA) at ×200 magnification.

### 2.9. BALF Analysis

To obtain BALF, an 18-gauge catheter was inserted into the trachea and perfused twice with 800 *µ*L of autoclaved PBS through the tracheal cannula as described previously [14]. Collected BALF was then centrifuged at 400 *g* for 10 min at 4°C, supernatant was transferred to fresh tubes, and pellets were resuspended in PBS. Total protein content in BALF was determined using a Bradford assay kit (Bio-Rad, Hercules, CA) according to the manufacturer's protocol. In brief, 10 *µ*L of BALF and 200 *µ*L of Bradford agent were added to a 96-well plate and incubated for 5 min at room temperature. The absorbance was measured at 595 nm using a spectrophotometer (Molecular Devices, Sunnyvale, CA). To count differential cells, pellets were resuspended in PBS, fixed onto slides, and stained using a Diff-Quik staining kit (Sysmex Co., Kobe, Japan). Total cell numbers in BALF were determined using a hemacytometer.

### 2.10. Statistical Analysis

Statistical analyses were performed in GraphPad 7 software (GraphPad Software Inc., San Diego, CA) using one-way analysis of variance followed by Dunnett's multiple comparisons test. *p* values <0.05 were considered statistically significant. The results were expressed as mean ± standard deviation (SD) for *in vitro* experiments and mean ± standard error of the mean (SEM) for *in vivo* experiments.

## 3. Results

### 3.1. Effects of EELCT on Inflammatory Mediators in Macrophages

**Figure 2:**
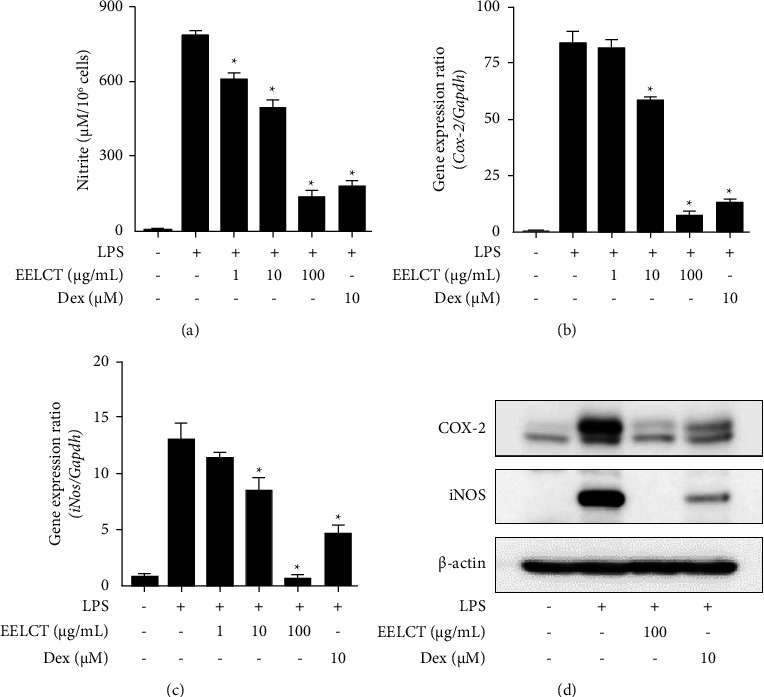
Effects of EELCT on NO production and COX-2 and iNOS expression in RAW 264.7 cells. (a) Cells were pretreated with/without drugs (EELCT or Dex) for 1 h and stimulated with LPS (100 ng/mL). After 24 h, NO production in culture media was determined using Griess reagent. (b, c) After 6 h, relative gene expression was verified by qPCR. (d) After 12 h, protein expression was determined by Western blot. Graph data represent mean ± SD. ^*∗*^*p* < 0.05 compared with the LPS-stimulated group. *β*-Actin was the loading control.

**Figure 3:**
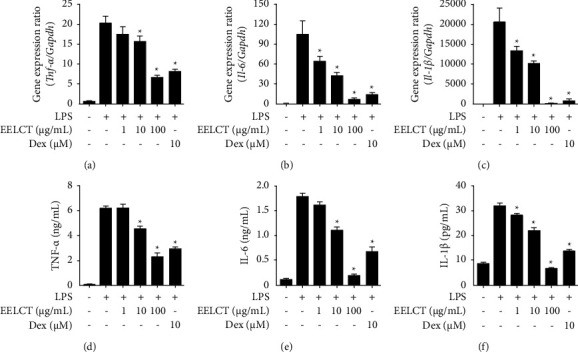
Effects of EELCT on inflammatory cytokine expression in RAW 264.7 cells. Cells were pretreated with/without drugs (EELCT or Dex) for 1 h and stimulated with LPS (100 ng/mL). (a–c) After 6 h, relative gene expression was quantified by qPCR. (d–f) After 12 h, inflammatory cytokine secretion was determined by ELISA. Graph data represent mean ± SD. ^*∗*^*p* < 0.05 compared with the LPS-stimulated group.

To investigate the mechanism action of traditional usage of *C. terniflora* on inflammation, *in vitro* studies were conducted using LPS-stimulated macrophages. First, to rule out the cytotoxicity of EELCT, 3-(4,5-dimethylthiazol-2-yl)-2,5-diphenyl-2H-tetrazolium bromide assays were performed. Cell viability was not affected up to 1,000 *μ*g/mL; therefore, we used below 100 *μ*g/mL EELCT in our study (Supplementary Figure S1). The various mediators from activated macrophages are closely involved in development of inflammation. From our data, EELCT significantly inhibited LPS-induced NO production in macrophages (Figure 2(a)). In this regard, expression of iNOS was also investigated. Our data showed that EELCT suppressed the gene and protein expression of COX-2, iNOS, TNF-*α*, IL-6, and IL-1*β* in a concentration-dependent manner (Figures 2(b)–2(d) and Figure 3).

### 3.2. Effects of EELCT on NF-*κ*B and MAPK Activation in Macrophages

**Figure 4:**
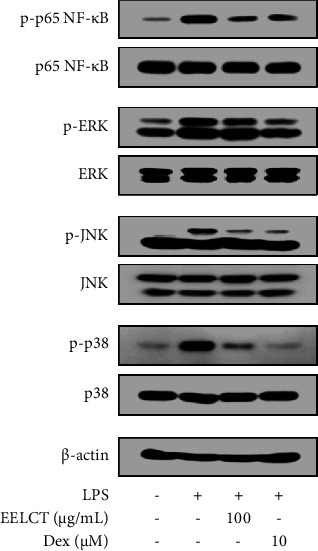
Effects of EELCT on NF-*κ*B and MAPK activation in RAW 264.7 cells. Cells were pretreated with/without drugs (EELCT or Dex) for 1 h and stimulated with LPS (100 ng/mL). After 15 min, total proteins were extracted and p65-NF-*κ*B, ERK, JNK, and p38 phosphorylation was assayed by Western blot. *β*-Actin was the loading control.

To identify signaling mechanisms of the EELCT action, NF-*κ*B and MAPK activation was assessed by determining phosphorylation of subfamilies. We observed that LPS-induced phosphorylation of p65-NF-*κ*B, extracellular signal-regulated kinase (ERK), c-Jun N-terminal kinase (JNK), and p38 was reduced by EELCT (Figure 4). Collectively, EELCT appeared to inhibit the expression of major inflammatory mediators, such as NO, COX-2, TNF-*α*, IL-6, IL-1*β*, and iNOS, through suppression of NF-*κ*B and MAPK activation in macrophages.

### 3.3. Effects of EELCT on Histology and Immune Cell Infiltration in ALI

**Figure 5:**
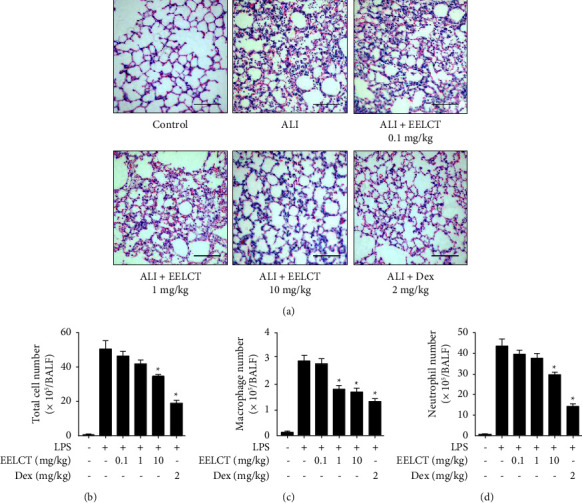
Effects of EELCT on histopathology and immune cell infiltration in ALI. Mice were intratracheally injected with LPS (5 mg/kg) before oral administration of EELCT or Dex. After 24 h, lung tissues and BALF were collected. (a) Representative images of H&E-stained lung sections (×200 magnification). (b–d) Diff-Quik-stained immune cell numbers in BALF. Scale bar = 100 *μ*m. Graph data represent mean ± SEM. ^*∗*^*p* < 0.05 compared with the ALI group.

To investigate the pharmacological effects of EELCT in the ALI model, mice were administrated EELCT or Dex and intratracheally injected with LPS (5 mg/kg). After 24 h, lung tissue and BALF were collected. Lung tissue was stained with H&E for histological analysis. In the ALI group, lung destruction, including immune cell infiltration, alveolar collapse, hemorrhage in the stroma, and airspace edema were observed, but these were alleviated in the EELCT-administrated group (Figure 5(a)). The immune cell infiltration was investigated by counting cell numbers in BALF. In our data, increased total cell numbers during ALI were reduced by oral administration of EELCT (Figure 5(b)). In addition, the infiltration of macrophages and neutrophils, which are important effector cells in ALI pathogenesis, was determined using Diff-Quik staining. As a result, both macrophage and neutrophil numbers were increased by LPS and decreased by EELCT in a dose-dependent manner (Figures 5(c) and 5(d)).

### 3.4. Effects of EELCT on Inflammatory Mediators and Pulmonary Edema in ALI

**Figure 6:**
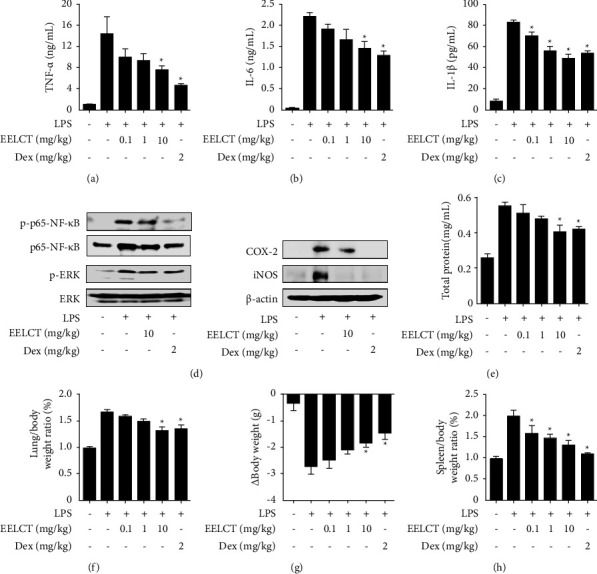
Effects of EELCT on inflammatory mediator production and pulmonary edema in ALI. Mice were intratracheally injected with LPS (5 mg/kg) before oral administration of EELCT or Dex. After 24 h, lung tissue and BALF were collected. (a–c) TNF-*α*, IL-6, and IL-1*β* levels in BALF were measured by ELISA. (d) NF-*κ*B, ERK, COX-2, and iNOS expressions in lung tissues were investigated using Western blot. (e) Total protein concentrations in BALF were determined using the Bradford assay kit. (f) Graph shows lung/body weight ratios. (g) Mice were weighed before EELCT or Dex administration and before sacrifice. The graph reveals the body weight change. (h) The graph represents spleen/body weight ratios. Graph data represent mean ± SEM. ^*∗*^*p* < 0.05 compared with the ALI group. *β*-Actin was the loading control.

To investigate the anti-inflammatory effect of EELCT during ALI, we evaluated TNF-*α*, IL-6, IL-1*β*, and MPO levels in BALF. Oral administration of EELCT dose-dependently suppressed the LPS-induced TNF-*α*, IL-6, IL-1*β*, and MPO production (Figures 6(a)–6(c) and Supplementary Figure S2). In addition, we confirmed the expression of important proteins in lung tissues. As a result, LPS increased the expression of NF-*κ*B, COX-2, and iNOS that reduced by the oral administration of EELCT. In addition, EELCT inhibited the phosphorylation of NF-*κ*B and MAPKs (ERK) induced by LPS (Figure 6(d)). Protein-rich pulmonary edema occurs from alveolar barrier dysfunction and is a major symptom of ALI [25]. To determine the extent of protein accumulation, total protein concentration in BALF was measured. The mouse body and lung were also weighed using precision balance (Ohaus, Parsippany, NJ) to investigate the effects of EELCT in edema. As a result, total protein levels and lung/body weight ratios were decreased by EELCT in the LPS-induced ALI model (Figures 6(e) and 6(f)). The body weight and spleen weight changes are important resulting factors of ALI. Our data showed that the body weight was reduced by approximately 30% in LPS-treated group compared with normal state, and it was alleviated by oral administration of EELCT (Figure 6(g)). EELCT also inhibited the increased spleen/body weight ratio during ALI (Figure 6(h)). These results indicated that oral administration of EELCT can attenuate the inflammatory symptoms and systemic damage during ALI.

## 4. Discussion

As mentioned above, *C. terniflora* leaf is a traditional herbal medicine to treat diverse inflammatory diseases. This study aimed to provide the scientific evidence for anti-inflammatory effect of *C. terniflora* leaf using activated macrophages. Macrophages are closely involved in inflammatory response by releasing various mediators, such as NO, COX-2, and inflammatory cytokines. Excessive NO production induces the overexpression of inflammatory cytokines that can conversely promote the NO production, forming a positive feedback cycle that causes inflammation. In addition, COX-2 has been implicated in several inflammatory diseases, such as asthma, arthritis, and cancer. In *in vitro* study, EELCT markedly inhibited LPS-induced high levels of NO, COX-2, TNF-*α*, IL-6, IL-1*β*, and iNOS in macrophages. LPS stimulates macrophages *via* toll-like receptor 4 leading to activation of NF-*κ*B, MAPKs, and other intracellular signaling pathways [26]. Upon macrophage stimulation, NF-*κ*B is phosphorylated and translocated to the nucleus following degradation of I*κ*B. In the nucleus, NF-*κ*B binds (as a transcription factor) to promoter region in target genes [27–29]. MAPKs are well-characterized signaling pathways related to inflammatory reaction in activated macrophages [30]. Indeed, it is well known that expression of inflammatory mediators mentioned above is regulated by NF-*κ*B and MAPKs. We confirmed that EELCT inhibited p65-NF-*κ*B, ERK, JNK, and p38 phosphorylation in LPS-stimulated macrophages. Taken together, these findings indicate that EELCT exerted anti-inflammatory effects by suppressing the expression of NO, COX-2, TNF-*α*, IL-6, IL-1*β*, and iNOS through regulation of NF-*κ*B and MAPK activation in activated macrophages.

ALI and its more severe form (ARDS) are life-threatening inflammatory diseases with high fatality rates [31]. However, there is no effective pharmacotherapy, and the ALI management remains in supportive care [32]. In recent years, considerable research has been conducted to develop new ALI treatment from natural products [16]. On the basis of the anti-inflammatory effect of EELCT, we investigated the therapeutic activity of EELCT against ALI. Multiple immune cells regulate the immune responses and the microenvironment in the lungs by releasing different inflammatory mediators [33, 34]. Notably, resident or recruited macrophages and neutrophils mainly contribute to pathogenesis of ALI. Alveolar macrophages have essential roles in initiating and maintaining the inflammatory responses. Additionally, activated neutrophils release several toxic mediators, which enhance tissue damage and increase vascular permeability and alveolar edema [35, 36]. These immune cells mediate inflammatory responses by releasing various inflammatory cytokines, such as TNF-*α*, IL-6, and IL-1*β* [37, 38]. These cytokines can promote other signaling cascade and directly lead to lung injury. TNF-*α* and IL-6 are major cytokines, which stimulate other cytokine and chemokine secretion and contribute to severity of ALI [39, 40]. Furthermore, IL-1*β* is an important early mediator in ALI and inflammatory conditions [41]. MPO is one of the main toxic mediators from neutrophils and leads to tissue disruption during pulmonary inflammation [42]. As mentioned earlier, NF-*κ*B is an important signaling molecule that regulates various inflammatory factors, ultimately participating in lung damage [43]. Accordingly, inhibition of this system can be a critical strategy for therapeutic capabilities in inflammation during ALI. In this study, oral administration of EELCT reduced the macrophage and neutrophil numbers in BALF in addition to the expression of inflammatory mediators during ALI. Therefore, our results suggest that EELCT suppressed inflammatory responses during ALI.

Infiltrated immune cells induce alveolar barrier dysfunction and microvascular permeability increase, which causes protein-rich pulmonary edema [25]. Once pulmonary edema fluid accumulates, it causes increased breathing and impairs gas exchange leading to hypoxemia and ultimately acute respiratory failure [44]. Our data showed that the accumulated protein amounts in lung and mouse lung/body weight ratios were increased in ALI group and decreased in EELCT-administrated group. Therefore, oral administration of EELCT reduced protein-rich pulmonary edema, suggesting that EELCT alleviated alveolar barrier dysfunction during ALI. In addition, ALI induces a reduction of body weight, which is known to be involved in ARDS clinical outcomes [45]. The spleen is the largest peripheral lymphoid organ and performs a wide range of immunological functions as the first line of defense against bacterial endotoxins [46]. In this regard, the increased spleen/body weight ratio is regarded as a resulting factor of ALI [47, 48]. In our results, the oral administration of EELCT attenuated the reduction of body weight and increased spleen/body weight ratio during ALI. Collectively, EELCT suppressed typical ALI symptoms, such as tissue damage, immune cell infiltration, production of inflammatory mediators, and pulmonary edema. Thus, this study suggested that EELCT has therapeutic effect against ALI.

A previous study reported that *Clematis* species contained many bioactive compounds, including triterpenoid saponins, flavonoids, coumarins, and alkaloids [2]. Our HPLC profile of EELCT implied that kaempferol-3-*O*-*β*-rutinoside and kaempferol-3-*O*-glucoside-7-*O*-rhamnoside were the major EELCT components (Figure 1). Kaempferol is a dietary flavonoid commonly existing in fruits, vegetables, and Chinese herbs, with distinct anti-inflammatory properties [49]. In addition, kaempferol has been proposed to prevent and treat inflammatory diseases including ALI [50–53]. A study identified that kaempferol-3-*O*-*β*-rutinoside, the most abundant compound in EELCT, suppressed the expression of inflammation-related mediators, such as iNOS, COX-2, TNF-*α*, and IL-6, through regulation of NF-*κ*B and MAPK pathways in LPS-stimulated RAW 264.7 cells [54]. Thus, the anti-inflammatory effects of EELCT confirmed in this study may be due to a combined effect of active compounds, including kaempferol, resulting in a final anti-inflammatory action.

## 5. Conclusion

In summary, EELCT inhibited several critical inflammatory mediators, such as NO, COX-2, TNF-*α*, IL-6, IL-1*β*, and iNOS, by suppressing the NF-*κ*B and MAPK activation in LPS-stimulated macrophages. Additionally, EELCT suppressed inflammatory response, including tissue damage, immune cell infiltration, production of inflammatory mediators, and protein-rich edema in an LPS-induced ALI model. Therefore, we propose that EELCT may function as a therapeutic candidate for ALI. However, more comprehensive research is needed for the practical use of EELCT as a pharmacotherapy.

## Data Availability

The data used and analyzed in this study are included within the article and the supplementary information files.

## Supplementary Materials

Supplementary Materials Supplementary material consists of the additional data and the detailed information of primer sequences and antibodies. Supplementary Figure S1: effects of EELCT on cell viability. Supplementary Figure S2: effect of EELCT on MPO production in LPS-induced ALI model. Supplementary Table S1: primer sequences used for qPCR. Supplementary Table S2: antibody information used in Western blot.
