# Supplementary material for: Anti-Inflammatory Effects of Clematis terniflora Leaf on Lipopolysaccharide-Induced Acute Lung Injury
Source: Evid Based Complement Alternat Med. 2024 Jan 9;2024:6653893. doi: 10.1155/2024/6653893 (PMC10791263; doi:10.1155/2024/6653893)
Supplement: Supplementary Materials — Supplementary material consists of the additional data and the detailed information of primer sequences and antibodies. Supplementary Figure S1: effects of EELCT on cell viability. Supplementary Figure S2: effect of EELCT on MPO production in LPS-induced ALI model. Supplementary Table S1: primer sequences used for qPCR. Supplementary Table S2: antibody information used in Western blot. [file 6653893.f1.zip › Supplementary material-revised.pdf]

Anti-inflammatory effects of *Clematis terniflora* leaf on lipopolysaccharide-induced acute lung injury

Supplementary Figure S1. Effects of EELCT on cell viability.

A

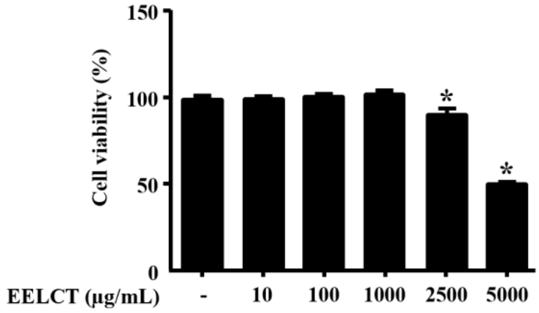

(A) RAW 264.7 cells (1 × 10^5 cells/well in a 96-well plate) were treated with/without EELCT (10–5,000 μg/mL) for 24 h and incubated with MTT reagent (1 mg/mL) for 2 h at 37 ºC. The formazan crystals were dissolved 100 μL of dimethyl sulfoxide and measured absorbance at 570 nm. Graph data represent the relative absorbance of cells treated with/ without EELCT as mean ± SD. *p < 0.05 compared with control cells.

Supplementary Figure S2. Effects of EELCT on MPO production in LPS-induced ALI model.

A

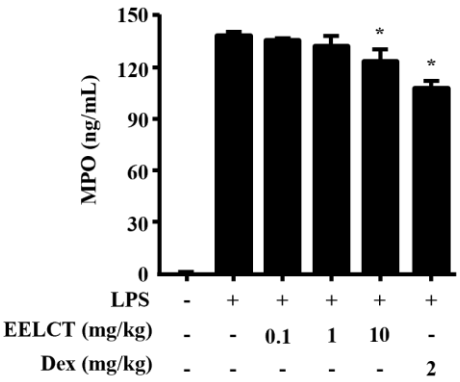

Mice were intratracheally injected with LPS (5 mg/kg) and 24 h later, mice were sacrificed and BALF were collected. (A) MPO levels in BALF was measured by ELISA. Graph data represent the mean ± SEM. *$p < 0.05$ compared with the ALI group. Dex: dexamethasone.

Supplementary Table S1. Primers sequences used for qPCR.

Table 1. Primer sequences used for qPCR.

Primer	Sequence 5'→3'	GenBank accession number
GAPDH	F: AGACACCATGGGGAAGGTGA<br>R: TGGAATTTGCCATGGGTGGA	NM_008084.3
COX-2	F: GCCAGGCTGAACTTCGAAACA<br>R: GCTCACGAGGCCACTGATACCTA	NM_011198.5
iNOS	F: GGAATGGAGACTGTCCCAGCA<br>R: GTCATGAGCAAAGGCGCAGA	NM_010927.4
TNF-α	F: GGCAGGTCTACTTTGGAGTCATTGC<br>R: ACATTCGAGGCTCCAGTGAATTCGG	NM_013693.3
IL-6	F: TCCAGTTGCCTTCTTGGGAC<br>R: GGTCTGTTGGGAGTGGTATC	NM_0.1168.2
IL-1β	F: GGACCTTCCAGGATGAGGAC<br>R: GTTCATCTCGGAGCCTGTAG	NM_008361.4

F, forward; R, reverse.

Supplementary Table S2. Antibody information used in Western blot.

Target	Supplier	Cat. No.	Size (kDa)	Host	Dilution
β-actin	Invitrogen	MA5-15739	42	Mouse	1:1000
COX-2	Santa Cruz	4842S	74	Rabbit	1:1000
iNOS	Cell Signaling	sc-651	130	Rabbit	1:1000
NF-κB p65	Cell Signaling	8242S	65	Rabbit	1:1000
p-NF-κB p65^{Ser536}	Cell Signaling	3033S	65	Rabbit	1:1000
ERK	Cell Signaling	9102S	42, 44	Rabbit	1:1000
p-ERK$^{Thr202/Tyr204}$	Cell Signaling	9101S	42, 44	Rabbit	1:1000
JNK	Cell Signaling	9252S	46, 54	Rabbit	1:1000
p-JNK$^{Thr183/Tyr185}$	Cell Signaling	9255S	46, 54	Mouse	1:1000
p38	Cell Signaling	9212S	38	Rabbit	1:1000
phospho-p38$^{Thr180/Tyr182}$	Cell Signaling	9215S	38	Rabbit	1:1000
Mouse IgG	Cell Signaling	7076S		Horse	1:2000
Rabbit IgG	Cell Signaling	7074S		Goat	1:2000

p, phospho
