# Supplementary material for: Anti-Inflammatory Effects of Clematis terniflora Leaf on Lipopolysaccharide-Induced Acute Lung Injury
Source: Evid Based Complement Alternat Med. 2024 Jan 9;2024:6653893. doi: 10.1155/2024/6653893 (PMC10791263; doi:10.1155/2024/6653893)
Supplement: Supplementary Materials — Supplementary material consists of the additional data and the detailed information of primer sequences and antibodies. Supplementary Figure S1: effects of EELCT on cell viability. Supplementary Figure S2: effect of EELCT on MPO production in LPS-induced ALI model. Supplementary Table S1: primer sequences used for qPCR. Supplementary Table S2: antibody information used in Western blot. [file 6653893.f1.zip › Table 1.docx]

Table 1. Primer sequences used for qPCR.

| Primer | Sequence 5'→3' | GenBank accession number |
| --- | --- | --- |
| GAPDH | F: AGACACCATGGGGAAGGTGA | NM_008084.3 |
|  | R: TGGAATTTGCCATGGGTGGA |  |
| COX-2 | F: GCCAGGCTGAACTTCGAAACA | NM_011198.5 |
|  | R: GCTCACGAGGCCACTGATACCTA |  |
| iNOS | F: GGAATGGAGACTGTCCCAGCA | NM_010927.4 |
|  | R: GTCATGAGCAAAGGCGCAGA |  |
| TNF-α | F: GGCAGGTCTACTTTGGAGTCATTGC | NM_013693.3 |
|  | R: ACATTCGAGGCTCCAGTGAATTCGG |  |
| IL-6 | F: TCCAGTTGCCTTCTTGGGAC | NM_0.1168.2 |
|  | R: GGTCTGTTGGGAGTGGTATC |  |
| IL-1β | F: GGACCTTCCAGGATGAGGAC | NM_008361.4 |
|  | R: GTTCATCTCGGAGCCTGTAG |  |

F, forward; R, reverse.
