# Supplementary material for: Anti-Inflammatory Effects of Clematis terniflora Leaf on Lipopolysaccharide-Induced Acute Lung Injury
Source: Evid Based Complement Alternat Med. 2024 Jan 9;2024:6653893. doi: 10.1155/2024/6653893 (PMC10791263; doi:10.1155/2024/6653893)
Supplement: Supplementary Materials — Supplementary material consists of the additional data and the detailed information of primer sequences and antibodies. Supplementary Figure S1: effects of EELCT on cell viability. Supplementary Figure S2: effect of EELCT on MPO production in LPS-induced ALI model. Supplementary Table S1: primer sequences used for qPCR. Supplementary Table S2: antibody information used in Western blot. [file 6653893.f1.zip › Table 2.docx]

Table 2. Antibodies used for Western blot.

| Target | Supplier | Cat. No. | Size (kDa) | Host | Dilution |
| --- | --- | --- | --- | --- | --- |
| β-actin | Invitrogen | MA5-15739 | 42 | Mouse | 1:1000 |
| COX-2 | Santa Cruz | 4842S | 74 | Rabbit | 1:1000 |
| iNOS | Cell Signaling | sc-651 | 130 | Rabbit | 1:1000 |
| NF-κB p65 | Cell Signaling | 8242S | 65 | Rabbit | 1:1000 |
| p-NF-κB p65^Ser536^ | Cell Signaling | 3033S | 65 | Rabbit | 1:1000 |
| ERK | Cell Signaling | 9102S | 42, 44 | Rabbit | 1:1000 |
| p-ERK^Thr202/Tyr204^ | Cell Signaling | 9101S | 42, 44 | Rabbit | 1:1000 |
| JNK | Cell Signaling | 9252S | 46, 54 | Rabbit | 1:1000 |
| p-JNK^Thr183/Tyr185^ | Cell Signaling | 9255S | 46, 54 | Mouse | 1:1000 |
| p38 | Cell Signaling | 9212S | 38 | Rabbit | 1:1000 |
| phospho-p38^Thr180/Tyr182^ | Cell Signaling | 9215S | 38 | Rabbit | 1:1000 |
| Mouse IgG | Cell Signaling | 7076S |  | Horse | 1:2000 |
| Rabbit IgG | Cell Signaling | 7074S |  | Goat | 1:2000 |

p, phospho
