# Supplementary material for: Anti-Inflammatory Effects of Clematis terniflora Leaf on Lipopolysaccharide-Induced Acute Lung Injury
Source: Evid Based Complement Alternat Med. 2024 Jan 9;2024:6653893. doi: 10.1155/2024/6653893 (PMC10791263; doi:10.1155/2024/6653893)
Supplement: Supplementary Materials — Supplementary material consists of the additional data and the detailed information of primer sequences and antibodies. Supplementary Figure S1: effects of EELCT on cell viability. Supplementary Figure S2: effect of EELCT on MPO production in LPS-induced ALI model. Supplementary Table S1: primer sequences used for qPCR. Supplementary Table S2: antibody information used in Western blot. [file 6653893.f1.zip › Supplementary material-revised.docx]

**Anti-inflammatory effects of *Clematis terniflora* leaf on lipopolysaccharide-induced acute lung injury**

**Supplementary Figure S1.** Effects of EELCT on cell viability.


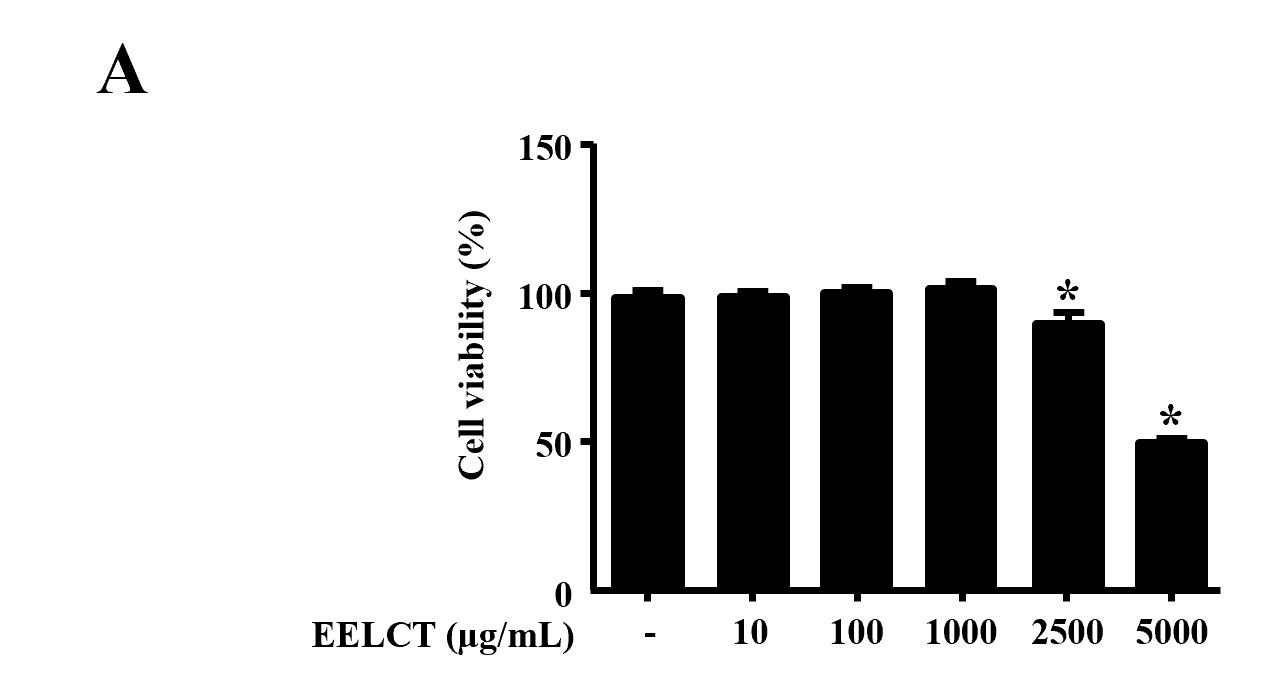


(A) RAW 264.7 cells (1 × 10^5^ cells/well in a 96-well plate) were treated with/without EELCT (10–5,000 µg/mL) for 24 h and incubated with MTT reagent (1 mg/mL) for 2 h at 37 ºC. The formazan crystals were dissolved 100 μL of dimethyl sulfoxide and measured absorbance at 570 nm. Graph data represent the relative absorbance of cells treated with/ without EELCT as mean ± SD. **p* < 0.05 compared with control cells.

**Supplementary Figure S2.** Effects of EELCT on MPO production in LPS-induced ALI model.

**
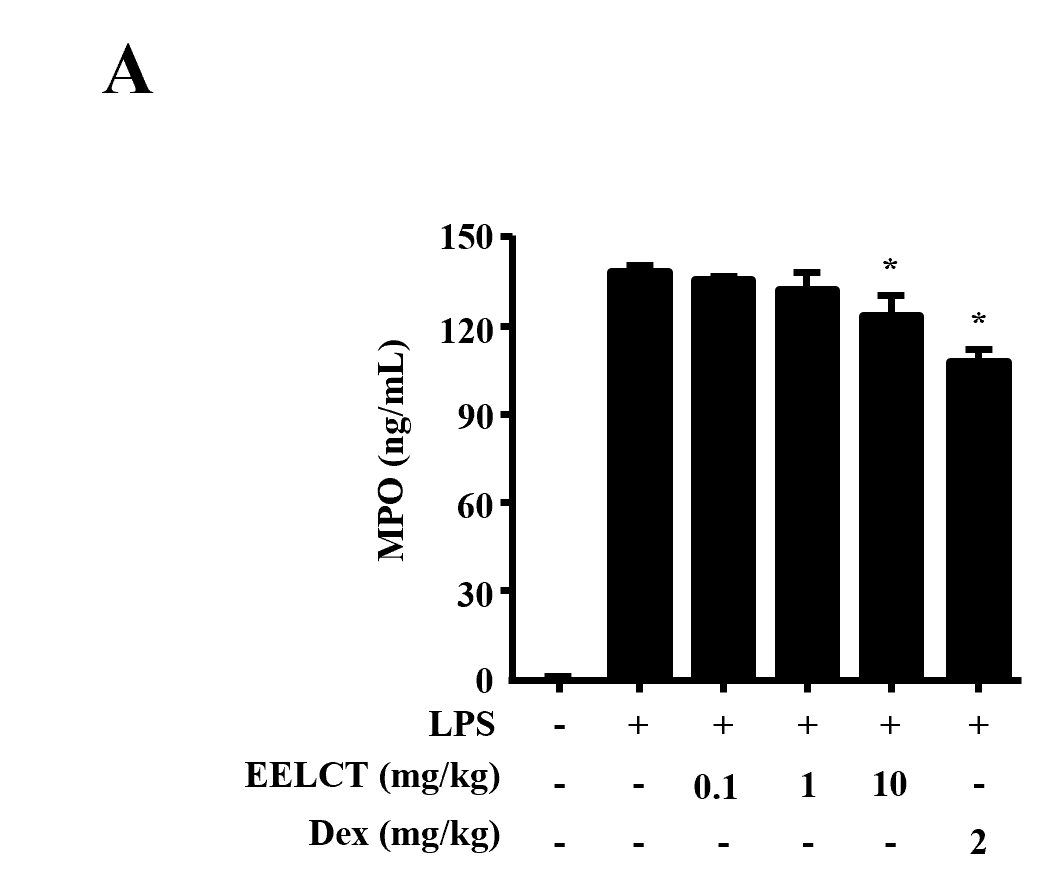
**

Mice were intratracheally injected with LPS (5 mg/kg) and 24 h later, mice were sacrificed and BALF were collected. (A) MPO levels in BALF was measured by ELISA. Graph data represent the mean ± SEM. **p* < 0.05 compared with the ALI group. Dex: dexamethasone.

**Supplementary Table S1.** Primers sequences used for qPCR.

**Supplementary Table S2.** Antibody information used in Western blot.
